# Hand-Book of Diseases of the Skin

**Published:** 1885-07

**Authors:** 


HAND-BOOK OF DISEASES OF THE SKIN—Edited by H. V Zeimssen, M. D., Professor of Clinical Medicine in Munich; Editor of Zeimssen's Cyclopedia of the Practice of Medicine. Illustrated with eighty wood engravings and color paints. New York: William Wood & Co. 1885.

This handsomely bound and valuable book of 658 pages is presented with the compliments of the publishers to every subscriber to Zeimssen's Cyclopedia. The book is made up with contributions from the following well-known authors: Anatomy and Development, by Paul G. Unna; Physiology, by H. Van Zeimssen; General Pathology and Therapeutics of the Skin, by Henrich Auspitz; Hyperdæmia, Æmia and Hemorrhages of the Skin, by Prof. Ernest Schwimmer; Dermatitis Superficiales, by Dr. T. H. Veiel; Deep Spreading Inflammations of the Skin—Acute, by Prof. E. Geber; Chronic, Deep-Spreading Inflammations, by Prof. Ernest Schwimmer; Anomalies of the Epidermis, Part I, by Dr. E. Lesser; Part II, by Dr. A. Weyl; Chronic Infectious Diseases of the Skin, by Prof. A. Neissen, M. D.; Neuroses of the Skin, by Prof. Ernest Schwimmer; Anomalies in the Growth and Color of the Hair, by Paul Michelson, M. D.; Anomalies in the Color of the Skin, by E. Lesser, M. D.; Anomalies of the Sebaceous Glands and their Function, by Ernest Veiel, M. D.; Acne Rosacæ and Sycosis, by T. H. Veiel, M. D.; Morbid Changes of the Nail and its Bed, by Prof. E. Geber, M. D.; Anomalies of the Sudoripharous Glands and their Functions, by Prof. E. Geber,M. D.; The Parasitic Diseases of the Skin, by A. Weyl, M. D., and Prof. E. Geber, M. D.; The New Formation of the Skin, by Prof. E. Schwimmer and V. Babes, M. D.; Neurima, Adenoma, Epithelioma, Malluscum and Carcinoma of the Skin, by Prof. E. Geber, M. D.

The book will prove invaluable to the general practitioner of medicine. The enterprising publishers are to be congratulated on the fine appearance of the volume.

